# Fat Embolism Syndrome: Evolving Perspectives on Diagnosis and Care

**DOI:** 10.7759/cureus.96136

**Published:** 2025-11-05

**Authors:** Nissar Shaikh, Bakri Alali, Umm E Amara, Umm E Nashrah, Naeem Alkheamy, Firdos Ummunnisa, Sayed Ghouri

**Affiliations:** 1 Surgical Intensive Care Medicine, Hamad Medical Corporation, Doha, QAT; 2 Clinical Academic Sciences, College of Medicine, Qatar University, Doha, QAT; 3 Anesthesia, Weill Cornell Medicine, Doha, QAT; 4 Obstetrics and Gynecology, Deccan College of Medical Sciences, Hyderabad, IND; 5 Medicine, Deccan College of Medical Sciences, Hyderabad, IND; 6 Medical Education, Hamad Medical Corporation, Doha, QAT; 7 Orthopedic Surgery, Hamad Medical Corporation, Doha, QAT

**Keywords:** acute respiratory distress, blindness, body contour surgery, cerebral fat embolization, fat embolism syndrome (fes), fat embolization syndrome, intramedullary nailing (imn), liposuction, neurological disturbances, ophthalmic fat embolism syndrome

## Abstract

Fat embolism syndrome (FES) is a rare clinical entity. Fat embolization in patients with trauma and long-bone fractures occurs in a majority of patients, and only a few patients develop the triad of skin petechia, respiratory distress, and neurological disturbances, which are termed FES. The exact incidence is not known, as there are no definitive criteria. All English-language publications about FES were searched and reviewed using Google Scholar, PubMed, various databases, conference proceedings, and abstracts, which were included in this study.

FES is particularly underdiagnosed and underreported in body contour plastic surgery patients. The following two main theories of FES pathophysiology are proposed: mechanical and biochemical. There are various risk factors for FES, mainly in young patients and bilateral femoral fractures. The diagnosis of FES was traditionally a diagnosis of exclusion. Recently, the combination of clinical parameters and imaging studies has increased the accuracy of FES diagnosis. Point-of-care ultrasound can show fat particles floating from the inferior vena cava to the right heart. MRI is more specific in diagnosing FES with brain involvement. A high index of suspicion, combined with early imaging studies and the application of clinical criteria, must be used to diagnose FES early and accurately. Pharmacological treatments, ranging from ethanol to aspirin, are used without any definitive recommendations. A recent meta-analysis showed the beneficial effect of steroids for FES. The early surgical intervention with open reduction and internal fixation appears to be a better choice, as conservative management and intramedullary nailing increase the risk of systemic fat embolization. The various types of bone reamers were not preventive of FES. The use of reamer irrigation and aspiration systems was found to decrease systemic fat embolization. With all the recent developments in acute and critical care, namely hydration, ventilation, and other resuscitative measures, the outcomes have improved. Blindness due to ophthalmic fat embolism syndrome is rarely reported. The mortality ranges from 5% to 15% in cases of FES.

## Introduction and background

Fat embolization occurs in the majority of patients with long-bone fractures, which is considered to be a benign and transient state. When this fat embolus causes organ dysfunction, mainly respiratory, neurological, and dermatological lesions, it is called fat embolization syndrome (FES), a rare scenario that can be potentially fatal. Famous Formula 1 driver Ronnie Peterson died after multiple long-bone fractures caused FES [1]. Von Bergmann clinically diagnosed FES for the first time in 1873 [2]. FES frequently occurs in long-bone fracture and pelvic trauma patients; it can also occur in medical conditions, such as post-bone marrow transplant, liposuction, osteomyelitis, or pancreatitis [3]. Early diagnosis is essential for managing FES; however, complications may still occur despite timely treatment. Recently, imaging studies have enhanced the diagnostic approach to fat embolism syndrome.

## Review

Epidemiology

The exact epidemiology of FES is unknown, as mild cases go unnoticed. The second reason for not diagnosing FES is that the clinical criteria are not specific. In earlier years (1960-1980), FES was diagnosed at autopsy, and the reported incidence was high. In recent years, a systematic review concluded that there has been a decreasing trend in FES. Furthermore, unilateral femur fractures were reported to have a 2.9% incidence of FES compared to pathological fractures (3.3%) and bilateral fractures (4.6%) [4]. Overall, the incidence varies from 1% to 29% [5]. Although Habashi et al. reported FES incidence up to 5% of all long-bone fractures [6]. Still, the actual incidence may not be known, as reports from the clinical criteria-based diagnosed FES mention an incidence of 0.9%. In contrast, the post-mortem-based studies reported the incidence of FES to be approximately 20% [7].

Pathophysiology

**Figure 1:**
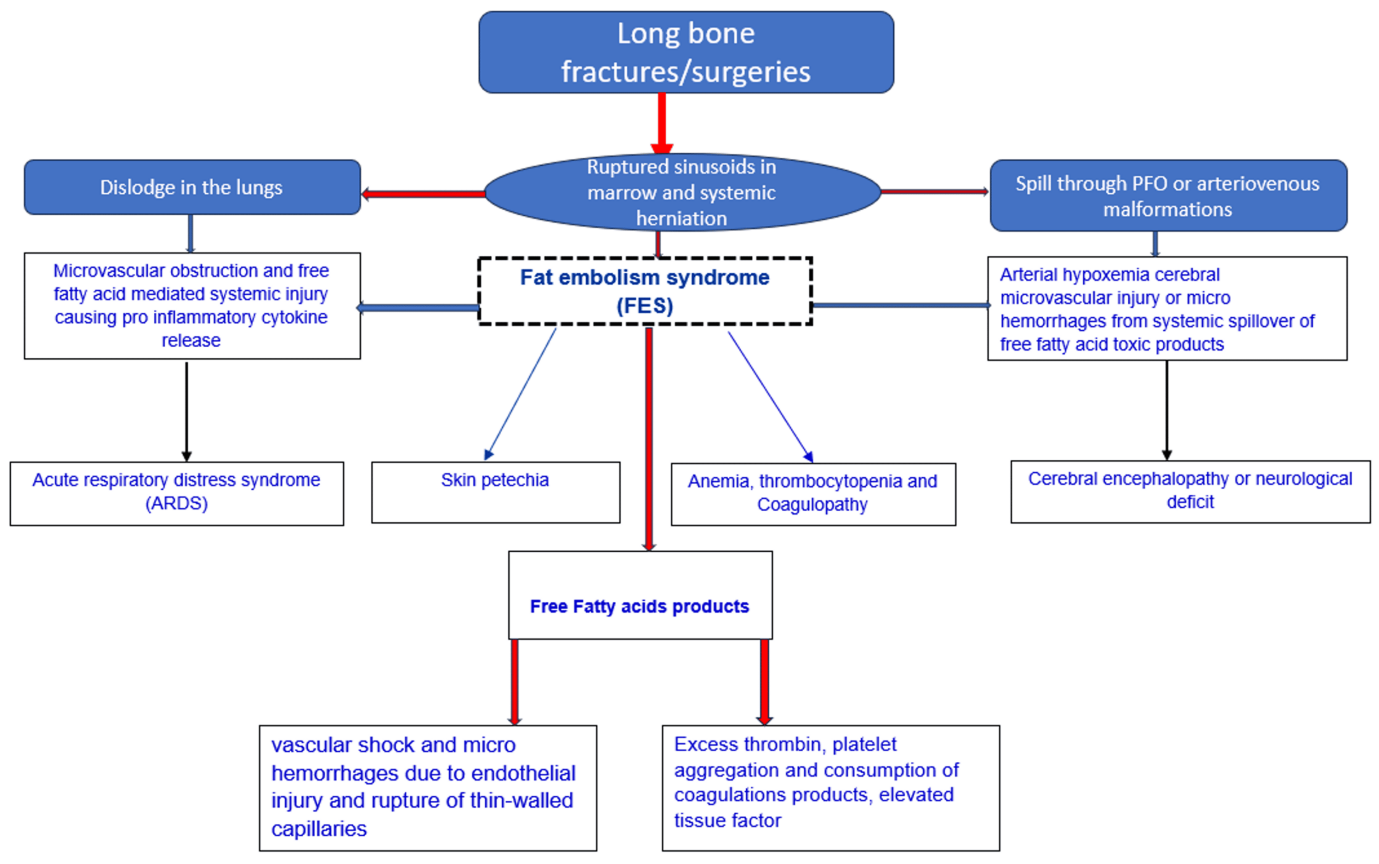
The pathophysiology of fat embolism syndrome. This image is created by the authors of this study. PFO: patent foramen ovale

The exact pathophysiology of FES is not well understood; however, two widely proposed theories explaining its occurrence are discussed further (Figure 1).

Mechanical Theory

Initially proposed by Gauss in the 19th century, the theory states that the first factor is the occurrence of adipose tissue injury, the second is the occurrence of various vascular injuries in secondary areas of fracture, and the third factor for FES is the passage of free fat globules from the exposed venules into the systemic circulation [8]. As per the mechanical theory for the occurrence of FES, most FES cases occur in long-bone fracture due to injury and opening of the venules in the bone marrow, and these venules remain open due to the osseous attachments, preventing these veins from collapsing and with the adipose tissue injury, the free fat globules enter through the open venules into the circulation and majority of these fat globules are filtered into the lungs. Fewer small super fat globules enter the arterial circulation, causing distal organ injury and dysfunction [9]. The fat globules have a flexible shape and become elongated to pass through the microcapillaries in the lungs. Another interesting factor contributing to the systemic spread of fat globules is arteriovenous malformation, which is due to hypoxia-induced arteriovenous (AV) malformation in the lungs [10]. Lastly, in up to 30% of adult populations, a persistent foramen ovale will remain patent. In hypoxia and lung injury, the fat globules are carried into the arterial circulation with elevated right heart pressure [11]. It is difficult to explain the 24-48 h delay of FES occurrence in non-surgical or trauma patients by this mechanical theory.

Biochemical Theory

Lehman and Moore proposed the chemical theory for FES. According to this, the production of trace particles of fatty acids, chylomicrons, low-density lipoproteins, and liposomes from the system releases fat molecules, leading to organ dysfunction [12]. This theory explains the occurrence of FES after 24-48 h of injury or the presence of risk factors. The free fatty acids cause an increase in the release of catecholamines and further lipolysis. The mobilization of excessive triglycerides leads to incomplete binding to albumin, allowing them to enter the circulation and cause distal organ dysfunction. This theory reports that natural fat is benign and does not cause organ injury (lungs). In contrast, hydrolyzed fat molecules and free fatty acids may cause lung injury, leading to acute respiratory distress syndrome (ARDS) [13].

Risk factors

The following table mentions the risk factors for traumatic and non-traumatic fat embolization syndrome (Table 1) [5,14].

**Table 1:** Risk factors for traumatic and non-traumatic fat embolism syndrome. IM: intramedullary

Traumatic fat embolism syndrome	Non-traumatic fat embolism syndrome
Young patients	Severe acute pancreatitis
Increase the gap between nail and bone	Lipid infusion
Close fracture, multiple fractures, bilateral femur fracture	Fatty liver
Pathological fractures	Liposuction
Overzealous IM nailing, reaming of long-bone fractures, and increased velocity of reaming	Bone marrow transplant
Delay in fracture fixation	-

Diagnosis of FES

Diagnosis of fat embolization syndrome is a vital pillar for patient outcomes. The diagnosis of FES based on clinical criteria is not specific, but recent developments in magnetic technologies have improved the diagnosis and management of FES (Tables 2-4) [5].

**Table 2:** Lindeque's criteria: FES can be diagnosed using respiratory criteria. FES: fat embolism syndrome

Lindeque’s criteria for the diagnosis of fat embolism syndrome
The partial pressure of oxygen (PaO_2_): <8 kPa
The partial pressure of carbon dioxide (PaCO_2_): <7.3 kPa
Tachypnea ≥35/min with sedation
Dyspnea, tachycardia, and anxiety

**Table 3:** Schonfeld's criteria for the diagnosis of fat embolism syndrome. The diagnosis of FES requires a cumulative score of >5 points. FES: fat embolism syndrome

Clinical manifestation	Number of points
Petechiae	5
Diffuse infiltrates on chest X-ray	4
Hypoxemia	3
Confusion	1
Fever	1
Tachycardia	1
Tachypnea	1

**Table 4:** Gurd and Wilson's criteria for the diagnosis of fat embolism syndrome. At least one major criterion and four minor criteria are required to diagnose FES. FES: fat embolism syndrome

Major criteria	Minor criteria
Respiratory distress	Tachycardia
Petechial rash	Fever
Cerebral disturbances	Retinal changes
-	Icterus
-	Renal impairment of lipiduria
-	Decrease in hemoglobin
-	Increased ESR
-	Fat macroglobinemia

Clinical Signs and Symptoms and Chest X-Ray

**Figure 2:**
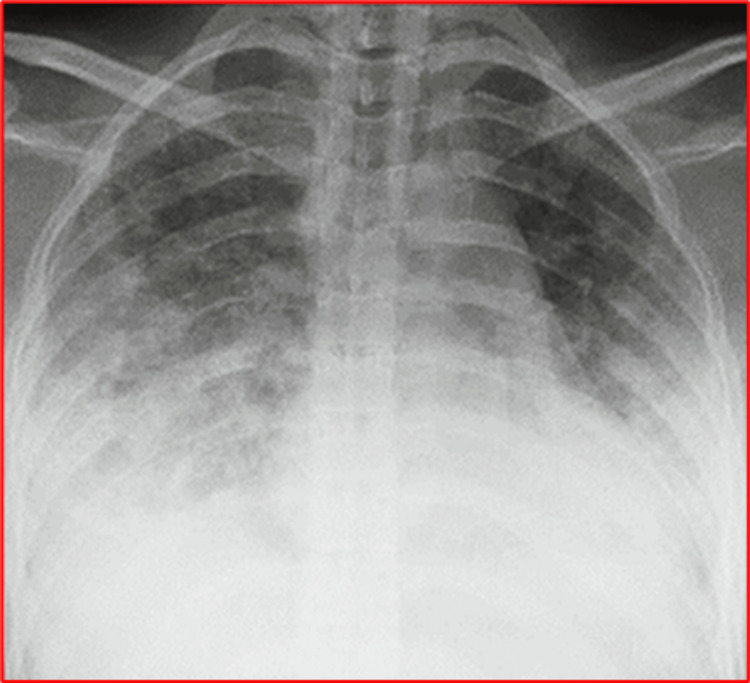
Chest X-ray showing diffuse infiltrates. This figure was obtained from Dr. Nissar Shaikh’s image collection; the patient's informed consent was obtained.

Imaging studies, such as chest X-ray, computerized tomography (CT), and MRI, will also help diagnose FES [15,16]. The chest X-ray findings in FES patients are non-specific and may be similar to the acute respiratory distress syndrome (ARDS), with normal heart size and the absence of septal lines or pleural effusion. The typical findings in FES are flake-like pulmonary shadows or snowstorm appearance (Figure 2).

Chest Computed Tomography (CT)

**Figure 3:**
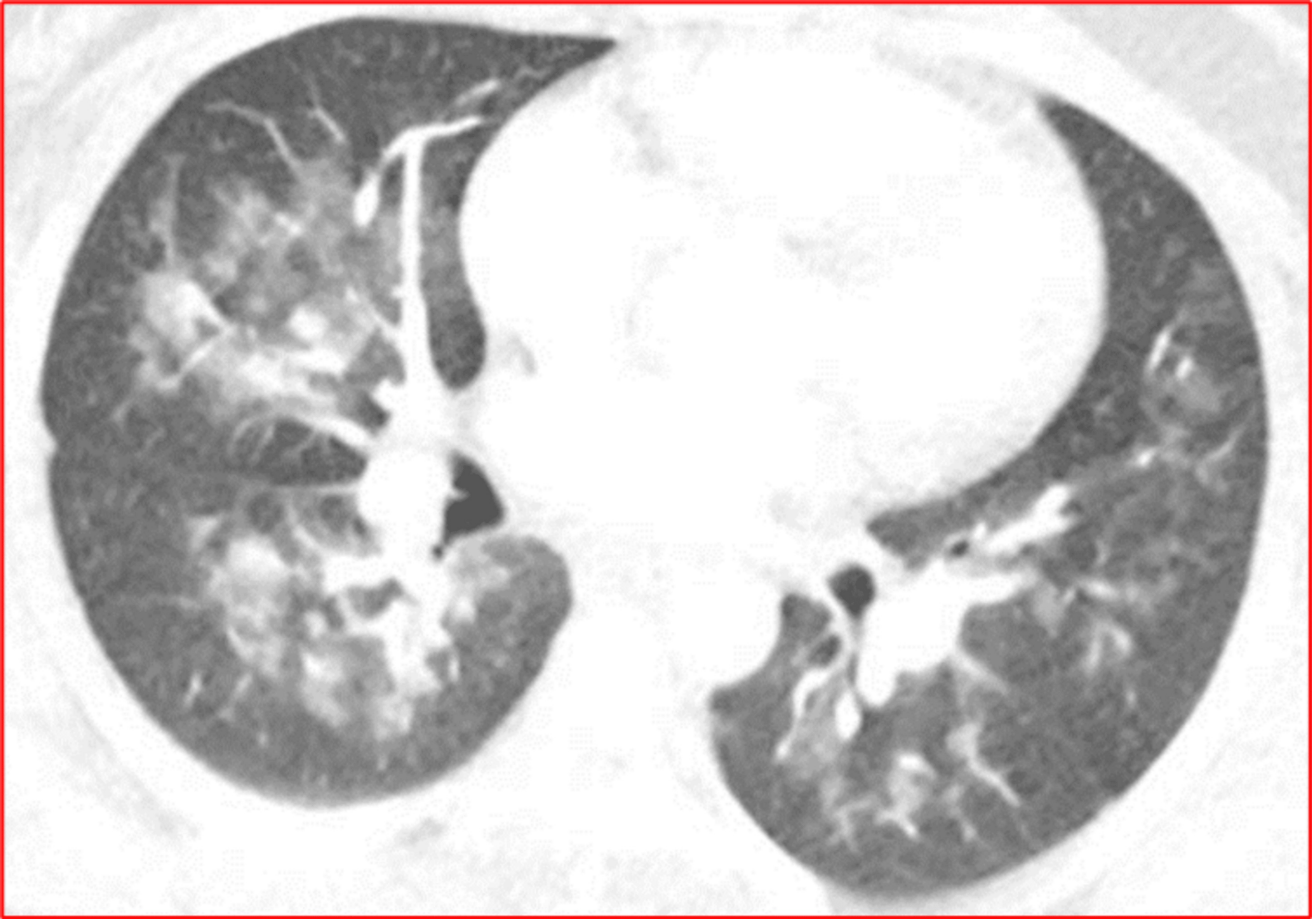
Typical CT chest findings in FES showing bilateral alveolar opacities. This figure was obtained from Dr. Nissar Shaikh’s image collection; the patient's informed consent was obtained. FES: fat embolism syndrome

Commonly reported findings on CT chest in patients with fat embolization syndrome, or even with only pulmonary fat embolism syndrome (fat embolization to the lungs only), include a geographic ground-glass appearance with or without septal thickening. Smaller nodules of variable sizes represent alveolar edema, hemorrhage, and inflamed lymph nodes secondary to fat embolization (Figure 3). Rarely in patients with non-luminant FES one can find fat-attenuating filling defects in the pulmonary arteries [17].

Magnetic Resonance Imaging (MRI) Brain

MRI is an indispensable tool for evaluating brain and spine pathology, and it is more sensitive in diagnosing cerebral fat embolism syndrome. The main concerns are MRI compatibility and the prevention of any retained metallic fragments that may move and heat up once the magnets are activated. The external fixators are metal pins or screws inserted into deeper tissues for stabilization and to prevent movement of the fractured bone. There are a few concerns about whether the material is ferromagnetic, which can cause internal tissue injury. Luckily, most external fixators are not ferromagnetic; hence, this problem is prevented. The fixator can generate heat, which can cause tissue injury, with the inner pins producing more heat and tissue damage. The third issue is whether the metal degraded the quality. The bulk of the literature concluded that heat effects are relatively small and decrease with increasing distance from the magnet. Non-ferromagnetic materials move very little, if at all, and modern internal fixators do not interfere with imaging.

External fixators have tremendous variability; one should check with handheld magnets and manufacturer recommendations. The following table shows the MRI compatibility of the external fixators according to the American Society for Testing and Materials (ASTM) International (Table 2) [5,18].

**Figure 4:**
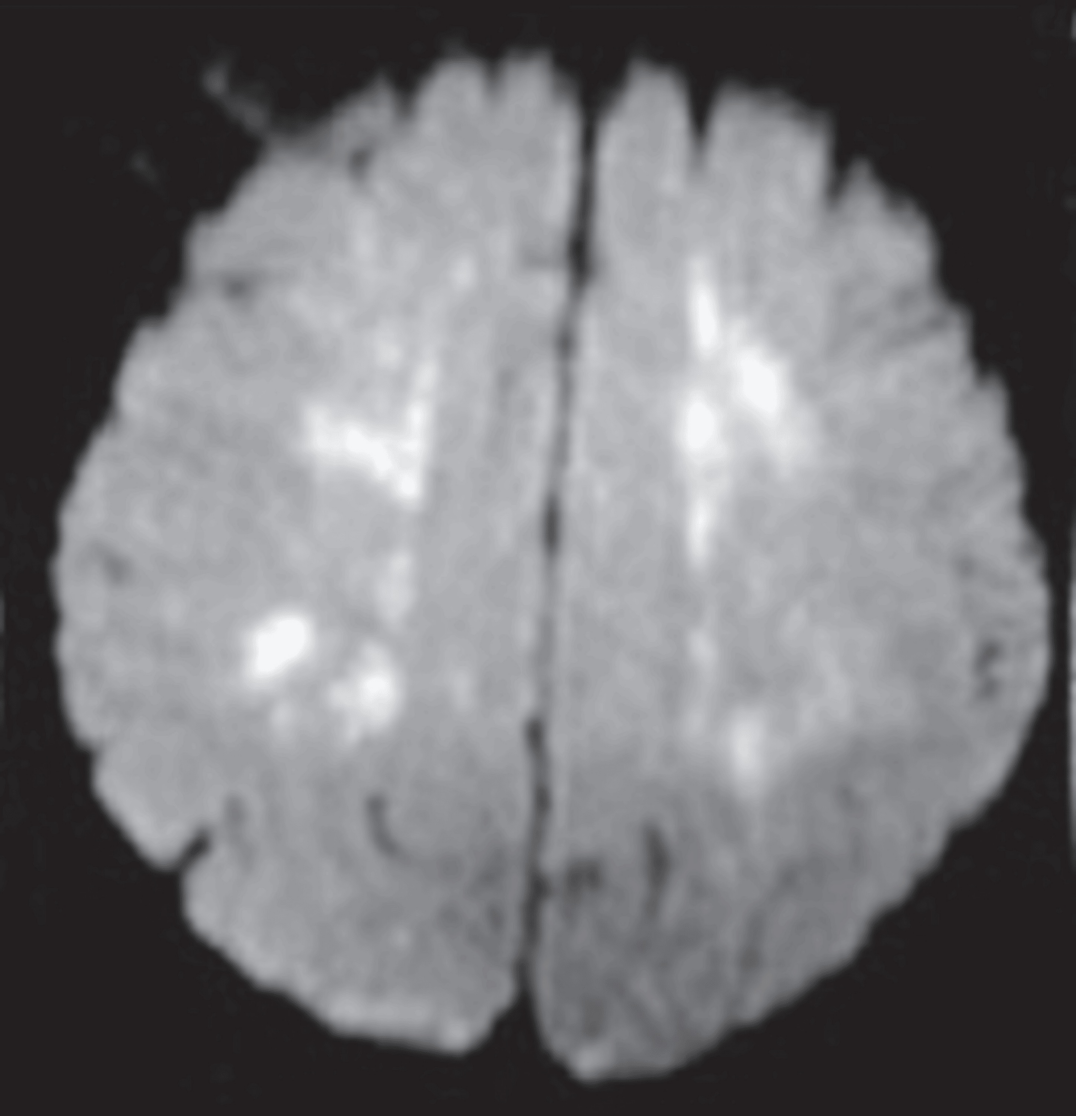
Starfield pattern of MRI brain in FES. This figure was obtained from Dr. Nissar Shaikh’s image collection; the patient's informed consent was obtained. FES: fat embolism syndrome

MRI findings are mainly due to macrofat embolism, which shows typical ischemic changes due to large vessel occlusion. The microfat embolism causes bilateral, symmetrical changes predominantly in the subcortical white matter, subcortical U-fibers, corpus callosum, and internal capsule. The disturbances of these minor ischemic areas depend on the severity of FES. In diffusion-weighted imaging (DWI), the picture differs in FES's early and late phases. From the first to fourth day, DWI will show a starfield pattern due to cytotoxic edema in the white matter (Figure 4). In the late phase, from the fifth to the 14th day, DWI shows the white matter's confluent areas of cytotoxic edema. Susceptibility-weighted imaging (SWI) will show pontine microhemorrhage in the white matter, also called the walnut kernel pattern [19].

According to MRI findings, cerebral fat embolism syndrome is classified into the following three types: (1) acute or type 1 - starfield, appearance due to cytotoxic edema; (2) subacute or type 2, which has the subtypes: type 2A - confluent cytotoxic edema in white matter, type 2B - diffuse vasogenic edema, type 2C - prehelical hemorrhage in white matter; and type 3is chronic with cerebral gliosis and atrophy [19].

Previous research has indicated a significant correlation between major and minor clinical criteria in fat embolism syndrome and abnormal imaging studies [15]. For instance, abnormal MRI findings were significantly associated with decreased consciousness level and oliguria [15].

Point-of-Care Ultrasound (POCUS)

Point-of-care ultrasound is an important, easy, non-invasive bedside tool that can be performed when FES is clinically suspected. POCUS may show several particles or floating white dots, mainly towards the right atrium from the inferior vena cava. Depending upon the severity, FES may impact the right side of the heart in severe cases with dilated right ventricle with tricuspid regurgitation [20].

Overall, imaging studies have a low specificity for diagnosing FES. Additionally, the timing of the imaging matters. For instance, the typical MRI findings of FES would be missed if the MRI is performed too early or too late. Bronchoscopic lavage and lipid samples are not specific, and fat globules are detected at the time of initial testing in more than 30% of FES cases [21].

Treatment

The management of fat embolism syndrome (FES) is primarily supportive and conservative, as no specific curative therapy currently exists. The main goals are to maintain adequate oxygenation, support hemodynamic stability, and prevent further embolic events.

Pharmacological Intervention

There are no specific treatments or pharmacological interventions for the treatment of FES. Supportive measures in FES include oxygenation, ventilation, maintaining hemodynamic stability, and management of physiological derangement. These supportive measures are implemented by ventilation, albumin-containing fluid resuscitation, external fixation or splinting of fractured bones, vasopressor support, temperature management, and advanced hemodynamic monitoring. The use of heparin or ethanol is not helpful. Steroid therapy in FES patients is proposed to be effective because it inhibits complement-activated leukocyte aggregation, thereby limiting free fatty acid levels and stabilizing membranes. It is reported that standard therapy decreases the risk of FES by 77% in patients with long-bone fractures. Conversely, steroid therapy does not impact infections, alveolar necrosis, or mortality [22,23]. Patients with FES are commonly dehydrated, and resuscitation with albumin has been reported to be beneficial as albumin binds with free fatty acids [16].

Surgical Intervention

Delayed fixation of long-bone fractures and conservative management increase the risk of FES. Intramedullary nailing, open reduction, and internal fixation are effective because they do not increase intermediate pressure and thus lower the risk of FES. Some techniques include lavage of bone marrow before fixation of long-bone fractures, venting the femoral bone, or drilling small holes in the bone cortex to decrease the intermediate pressure.

In the 1950s and 1960s, early fixation of long-bone fractures was blamed for the occurrence of FES. In the 1980s, the concept of patients being too sick to be operated on had delayed the fixation of long-bone fractures, thus causing FES. All the modern trauma care concepts with fixation of the long-bone fractures in 24-72 h are beneficial in preventing the occurrence of FES [4]. For those patients requiring intermediary nail fixation of long-bone fractures, the modified reamer systems, like slim reamer, sharp hip reamer, and enlarged reamer flute, were compared with nailing without reaming of the bone canal to reduce the intramedullary pressure and reduction in fat embolization; however, there were no significant differences with respect to the patient outcome [24].

The reamer irrigation aspiration (RIA) system was also developed, allowing for the suction of intermediary content to reduce systemic fat embolization during reaming of the bone canal. RIA effectively reduces fat embolic load into the circulation [25]. Additionally, RIA has been reported to be effective in preventing FES in elderly patients [26]. During reaming, the venting of bone creates an opening to relieve pressure and facilitates a decrease in intramedullary pressure, thus reducing systemic embolization of bone marrow fat globules. However, the evidence is not strong, so it is not recommended [27]. Overall, the resuscitative and supportive care of FES includes hydration, adequate nutrition, airway protection, oxygenation, and supportive ventilation, as well as maintaining hemodynamics and thromboembolism prophylaxis.

FES in body contour plastic surgery

There is an exponential increase in plastic body contour surgeries, particularly liposuction, lipofilling, and dermolipectomy, due to increased awareness and availability. From 2000 to 2016, there was a 132% increase in plastic surgical procedures [28]. Although the incidence of FES is rare in body contour surgeries, but with this rapid increase in the number of these surgeries, the incidence of FES will increase. Still, the FES is underdiagnosed and underreported. The literature on FES in body contouring surgeries is mainly comprised of case reports and small case series [29]. The risk factors for FES in body contour plastic surgeries include a large volume of lipoaspirate and combining procedures or performing more than one procedure in a single setting [30,31]. Kao et al. reviewed published cases of pulmonary fat embolism following liposuction and fat grafting, including a total of 38 cases. CT chest was helpful in diagnosing the FES in 100% of these cases; the five patients who died in their study were reported to have developed FES within 24 h of the surgery [32]. Instead of wasting time in excluding the other frequent causes of post-operative complications, a high index of suspicion in combination with the use of clinical criteria and early imaging studies will diagnose the FES in these plastic surgical patients.

Cerebral fat embolism syndrome

Cerebral fat embolism syndrome (CFES) is a rare, potentially fatal condition where the fat emboli enter the bloodstream, either after long-bone fractures or body contour plastic surgeries, causing neurological complications [33]. Symptoms typically appear from 12 to 72 h after the injury and range from confusion to coma, seizures, and or focal neurological deficits. Diagnosis is primarily based on a high index of suspicion, combined with clinical criteria supported by brain MRI, which may show a "starfield pattern" of lesions [34]. In pathogenesis, the primary trigger is trauma, long-bone fractures, or body-contour plastic surgery, leading to the dislodgement of marrow fat into the circulation. These fat globules travel through the bloodstream. They enter the arterial circulation directly through microglobules filtering through lung capillaries or indirectly via intracardiac shunts, such as a patent foramen ovale (PFO) [35]. The fat emboli lodge in the small blood vessels of the brain, leading to inflammation and a range of neurological issues. The therapy includes (a) supportive care, which is the cornerstone of treatment, and aggressive hemodynamic and respiratory support to manage neurological and respiratory complications [36], and (b) surgical Intervention and early fixation of orthopedic fractures (within the first 24 h), which is recommended to prevent further emboli and reduce the incidence and severity of CFES. Other therapies, such as dehydrating agents, head cooling, and other treatments, have been explored, but supportive care remains the most effective approach. The CFES outcome varies, but with supportive care and early interventions, the neurological manifestations of cerebral fat embolism tend to be reversible, and overall mortality can be low. Armstrong et al. reported a mortality of 20.5% in CFES. Early onset and a higher Takahashi grade on brain MRI are associated with higher mortality. The majority of patients improve, and only 3.7% of patients have cognitive dysfunctions that recover to normal neurological status from two to 13 weeks [37].

Ophthalmic fat embolism syndrome

Ophthalmic fat embolism syndrome is the appearance of vision-related symptoms in FES patients, a serious condition caused by fat globules traveling through the bloodstream to lodge in small blood vessels. Ophthalmic involvement, presenting with retinal hemorrhages, retinal whitening (Purtscher-like lesions), cotton wool spots, vision loss, or visual disturbances, is uncommon and often occurs without a cardiac defect, typically as a manifestation of systemic FES following a long-bone fracture or orthopedic trauma. Although rare, the ophthalmic fat embolism syndrome may occur 10 days after trauma or surgery. The diagnosis requires careful ophthalmoscopy, and while the visual symptoms can be variable, even blindness has been reported [38]. Early diagnosis and supportive therapy are crucial for recovery [39].

Prognosis

Modern medical and technological advances have led to a better and earlier diagnosis of FES. The advanced care in intensive therapy units has also improved the outcome of FES by reducing morbidity. The reported mortality in fat embolism syndrome ranges from 10% to 15%. Elderly patients, non-orthopedic etiologies for FES, and cerebral and respiratory complications of FES were significantly associated with mortality. Steroids have been shown to have beneficial effects and reduce mortality in patients with FES [40]. The prognosis of FES differs significantly between young and elderly patients; elderly patients are 24 times more likely to die in the hospital compared to younger patients under 40 years of age [41].

Differential diagnosis

FES should be differentiated from other common causes of skin rash, acute respiratory distress, and neurological disturbances. Particularly in FES, cerebral fat embolism syndrome should be differentiated from diffuse axonal injury on imaging studies. The cerebral petechial hemorrhages in FES are mainly in the white matter, whereas the diffuse external injury involves the frontotemporal lobes' grey and white matter interface. Additionally, patients with FES frequently have long-bone fractures, and they would be awake at presentation without lucid intervals. In contrast, others with diffuse axonal injury (DAI) have a decreased level of consciousness from the beginning. The DAI will also show abnormalities with hyperintense signals in both DWI and T2-flare images [42]. The differential diagnosis and differentiating conditions of FES are illustrated in Table 5.

**Table 5:** The differential diagnosis of FES. FES: fat embolism syndrome; CTPA: computerized tomographic pulmonary angiogram

Diagnosis	Differentiating conditions	Fat embolism finding
Pulmonary embolism	CTPA will show filling defect in pulmonary arteries	CTPA is normal in routine settings
Aspiration pneumonia	Decrease in the level of consciousness. Bronchoscopy will detect particles or aspirate. Tree-in-bud opacities. Frequent on right side	No history of altered consciousness. Bronchoscopy is normal; rarely, the far stain will be positive from the aspirate. Frequent in upper lobes
Pulmonary contusion	Develops shortly and commonly within 6 h of trauma. Localize or multifocal	24-48 h after trauma or surgery. Frequently in upper or dependent lobes
Pulmonary edema	Bilateral diffuse infiltrates risk factors	Asymmetrical and patchy infiltrates are risk factors
Pneumonia	Increased septic markers (procalcitonin)	Septic marker or procalcitonin is normal

## Conclusions

Fat embolization occurs in most patients undergoing long-bone fracture and body contour surgery. Only a few patients develop skin petechiae, respiratory distress, and neurological disturbances, collectively known as FES. The exact incidence is unknown, but it is widely reported to occur at 10-29%, depending on the timing, nature of the study, and criteria used. FES in plastic body contour surgeries is underreported and may be underdiagnosed.

The clinical criteria, such as those proposed by Gurd and Wilson, Lindeque, or Starfield, are non-specific but are still frequently used. More recently, the combination of CT chest and MRI brain, along with clinical criteria, has increased the specificity of FES diagnosis. Point-of-care ultrasound (POCUS) (bedside ultrasound) can show the fat particles in the inferior vena cava (IVC) and right heart. A high index of suspicion, combined with clinical criteria and imaging studies, will facilitate early diagnosis of FES. FES should be differentiated from DAI. In the initial resuscitation, albumin was reported to have a chelating effect on free fatty acids. Steroids were found to be effective in preventing FES in most cases. Initial external fixation or earlier open reduction and internal fixation are recommended, as intramedullary nailing has been associated with an increased risk of FES. Various surgical techniques, such as creating a small opening in the cortex and employing different types of reaming, were not effective in preventing FES. The remaining irrigation and aspiration systems look promising. FES and ophthalmic fat embolism syndrome can cause blindness. The majority of FES and cerebral fat embolism syndrome patients will improve and return to normal status, and around 3% of patients will have cognitive dysfunctions for up to 12 weeks or longer. Advances in intensive care have improved supportive care and outcomes for FES patients. Early diagnosis and multidisciplinary management in the intensive care therapy unit will improve prognosis.
